# Guanine crystal formation by the unicellular organism *Phacotus lenticularis* is part of a cellular stress response

**DOI:** 10.1371/journal.pone.0316193

**Published:** 2025-02-12

**Authors:** Noy Shaked, Andrea Sorrentino, Neta Varsano, Sefi Addadi, Ziv Porat, Iddo Pinkas, Steve Weiner, Lia Addadi

**Affiliations:** 1 Department of Chemical and Structural Biology, Weizmann Institute of Science, Rehovot, Israel; 2 MISTRAL Beamline, Experiments Division, ALBA Synchrotron Light Source, Cerdanyola del Valles, Barcelona, Spain; 3 Department of Chemical Research Support, Weizmann Institute of Science, Rehovot, Israel; 4 Department of Life Sciences Core Facilities, Weizmann Institute of Science, Rehovot, Israel; Sathyabama Institute of Science and Technology, INDIA

## Abstract

Organic crystals, and in particular guanine crystals, are widely used by multicellular organisms for manipulating light and producing structural colors. Many single celled eukaryotic organisms also produce organic crystals, and guanine is the most abundant type produced. Their functions are thought to be related to the fact that guanine is nitrogen rich. Here we studied a freshwater unicellular eukaryotic alga, *Phacotus lenticularis*, and found that when the growth medium is depleted in phosphorus, the alga stops reproducing and produces intracellular birefringent particles inside vesicles. Cryo-SEM showed that these particles are faceted and are located within membranes inside the cell. Using Raman spectroscopy, we showed that these particles are β-guanine crystals. 3D tomograms produced using cryo-soft-X-ray-microscopy quantitatively documented the increase in cell volume and distribution of guanine crystals within the cells with increasing time of phosphorous deprivation. The tomograms also showed additional morphological changes in other cellular organelles, namely starch granules, chloroplasts, nuclear DNA and membranes. The combined observations all indicate that under phosphorous depletion, the algal cells undergo a massive stress response. As guanine crystal formation is part of this response, we conclude that guanine crystals are formed in response to stress, and this is not related to nitrogen availability. Upon addition of phosphate to the P-depleted media, the algal cells, with their guanine crystals, resume reproduction. From this we conclude that the guanine crystals somehow contribute to the recovery from stress.

## Introduction

Guanine crystals are well documented in multicellular organisms [1–3]. They were first discovered in 1861 in fish scales, where they are responsible for the silvery iridescence of the scales and skin in many fish species [4]. Guanine crystals, as well as other structurally related organic crystals, have since been identified as components of tissues that produce structural colors (e.g. in chameleons [5], fish and cephalopods [6], certain copepods [7, 8], spiders [9], as well as in the eyes of certain invertebrates (e.g. arthropods [10], bivalves [11–13]) and vertebrates (e.g. fish [14–17]). In all these cases, the crystals are involved in manipulating light [18]. This is certainly related to the fact that these organic crystals have unusually high refractive indices in at least one optical direction [9].

Guanine crystals are also present in certain marine dinoflagellates, which are unicellular eukaryotic organisms [19, 20]. Because the dinoflagellates initially studied were bioluminescent, the guanine crystals were thought to be related to bioluminescence [19, 20]. However, when guanine crystals were also found in non-luminescent species, it was suggested that they function as waste products of nitrogen metabolism or for storage of nitrogen [21, 22]. Guanine crystals were also documented in another dinoflagellate species [23], and based on the 3D distribution of the guanine crystal-containing vacuoles in the cell, it was proposed that their functions might be related to enhancing photosynthesis by back reflecting stray light into the chloroplasts [23]. Guanine crystals were identified in two species of unicellular microalgae that are unrelated to dinoflagellates [24]. The authors tentatively suggested that the function of these guanine crystals might be related to nitrogen storage [24]. Recently, Pilátová et al. [25] surveyed more than 200 species of unicellular eukaryotic marine organisms from many different phyla by Raman microscopy, and found that 77% contained organic crystal inclusions, and of these 80% of the crystals were guanine. This seminal study shows that organic crystals are formed by many different unicellular eukaryotes. Pilátová et al. concluded that these nitrogen-rich organic crystals may have evolved as a solution for nitrogen detoxification, and/or as protection against exposure to high ammonia or nitrate concentrations.

Here we studied the unicellular organism *Phacotus lenticularis* from the *Chlamydomonadales* order. *P*. *lenticularis* is a green alga that is abundant in freshwater lakes and ponds worldwide [26]. *P*. *lenticularis* produces a crystalline calcitic shell [27, 28]. In fact, the shells can be a major constituent of the freshwater basin sediments and can influence carbonate influx and ion availability in the water [29–31]. While studying *P*. *lenticularis* calcite crystallization [32], we discovered that this freshwater unicellular alga can also produce guanine crystals when the cells are grown in a phosphate-depleted (P-depleted) medium. Here we document this phenomenon and propose that *P*. *lenticularis* forms guanine crystals in response to stress, which in this case is caused by limited phosphorus availability.

## Materials and methods

### Culturing *P*. *lenticularis* in artificial media

Three *P*. *lenticularis* isolated strains were purchased from the Culture Collection of Algae at Göttingen University (SAG) in Germany: strain 61–1 was isolated in the United Kingdom, and strains 15.99 and 16.99 were isolated from the German lakes Tollensee and Schulzensee, respectively. All strains were cultured in closed 50 ml flasks with membrane caps (Greiner Bio-one) and in culture volumes of 10 ml. Cultures were grown in a cooling incubator (INNOVA 4230) at controlled temperature of 20°C and with a day:night cycle of 18:6h. Philips LED lamp (100 W, 4000 K, 1521 lm) was used to provide constant growing conditions. The cultures were grown in two types of artificial media, as specified below:

MiEB_12_ is a generic medium commonly used for algae culturing at the SAG algae collection [33, 34]. The three *P*. *lenticularis* strains purchased from SAG were cultured in this medium for the last 30 years or more. MiEB_12_ contains high concentrations of nutrients and μ-nutrients, but it does not contain sufficient Ca or CO_3_ ions to produce CaCO_3_ saturation. Thus, MiEB_12_ media does not promote the formation of shells in *P*. *lenticularis* cultures and is used for the preservation of viable strains and non-calcified controls in the experiments. We used this medium for maintaining our cultures.

N-HS-Ca is a specific medium that was created especially for the culturing of *P*. *lenticularis* in previous studies [35]. This medium was designed to simulate the chemical conditions of hard-water lakes during *P*. *lenticularis* bloom. This medium was based on a previous *Phacotus*-tailored medium, N-HS, whose composition reflected the natural conditions in two German lakes, where *P*. *lenticularis* was found to be most abundant during its bloom period [36]. N-HS-Ca contains a higher concentration of calcium ions and CO_3_ derivatives which contribute to the stabilization of a high pH value of 9. N-HS-Ca medium was prepared in our lab according to Schlegel et al [35]. Briefly, three solutions were made by the dissolution of salts in DDW–bulk solution, micronutrient solution and vitamin solution. The bulk solution was formed by dissolving 7 salts ‐ CaSO_4_, (MgCO_3_)_4_·Mg(OH)_2_·5H_2_O, NaHCO_3_, CaCl_2_, Na_2_SiO_3_·5H_2_O, Ca(NO_3_)_2_·4H_2_O, and K_2_HPO_4_ in DDW as described in Schlegel et al [35]. The final concentrations of major ions in the bulk solution (in mM) were as follows: Ca^+2^: 1.9; Mg^+2^:0.4; K^+^: 0.1; NO_3_^−^: 1.5; PO_4_^−3^: 0.05; CO3^−2^: 0.08; HCO^3−^: 4.29.

The micronutrient solution contained 8 additional salts and the vitamin solution contained vitamin B_12_, biotin, thiamine HCl and nicotinamide. These were all dissolved in DDW and added to the bulk solution as described in Schlegel et al [35]. The prepared medium was filtered under sterile conditions before use.

In our experiments, we also grew the cultures in the N-HS-Ca medium without any K_2_HPO_4_, which we refer to as the P-depleted medium. Normal N-HS-Ca medium cultures in exponential growth were washed 3 times using centrifugation in P-depleted medium (5 min, 310 rcf). The cultures were then transferred into closed 50ml flasks with membrane caps (Greiner Bio-one) or 35 mm Petri dish with a glass bottom (D35-14-1.5-N, Cellvis or P35G-1.5-14.C, MatTek). P-depleted medium was added to half of the cultures, and the normal N-HS-Ca medium was added to the other half of the cultures as a control. Each experiment included 3–5 repeats of each condition (P-depleted and control) and 12 experiments were performed over the course of 2 years.

In the final experiment, we added back phosphate to the P-depleted culture, by preparing a concentrated stock solution of K_2_PO_4_, and then adding back an appropriate small volume, so that the final concentration of phosphate was 0.05mM as it is in the N-HS-Ca medium. We did not centrifuge and then add the phosphate solution so as to maintain the same growth conditions as in the control. Note too that to ensure that the depletion of K^+^ does not affect the cellular response, KNO_3_ was also added to the P-depleted medium to produce the same K^+^ concentration as the bulk medium in some of the experiments. No difference was observed.

As *P*. *lenticularis* is not a model organism, and N-HS-Ca is not a standard medium, we needed to establish growth curves to determine when the cultures were in exponential growth. Therefore, growth was quantitatively monitored by using an imaging flow cytometer (ImageStream X Mark II, AMNIS corp.–part of Cytek, CA, USA). The cells were imaged using a 60X lens (NA = 0.9). Lasers used were 405nm (2mW) for chlorophyll excitation and 785nm for side scatter collection. The channels acquired were brightfield (Ch09), side scatter (Ch06) and Chlorophyll (Ch11). Data were analyzed using IDEAS 6.3 (Amnis Corp.). Only single cells that contain chlorophyll (determined according to the intensity and area of chlorophyll staining) were used for analysis.

The experiment included 3 repeats of each condition and reproduced more than 10 times over the course of 2 years.

All cultures were monitored using several techniques:

### Light microscopy

*P*. *lenticularis* cells were imaged using a Nikon ECLIPSE E600 POL light microscope using an integrated Nikon digital sight camera under bright field illumination. An integrated cross-polarizer was used for the detection of birefringence, which shows the presence of crystalline components in the cells.

### Time-lapse experiment

To monitor the algal cell response to P-recovery, and to avoid any response of the cells to harvesting manipulations, K_2_PO_4_ from a concentrated solution was added to the cultures grown in P-depleted medium and the concentrated solution was gently pipetted into the culture just before data acquisition began.

#### Data acquisition

A glass-bottom Petri dish was mounted on a widefield Leica DMi8 inverted microscope (Leica-microsystems CMS GmbH, Germany), equipped with a monochromatic camera (Leica DFC7000GT) and integrated analyzer and polarizer lenses. Imaging was performed using a water immersion lens (HC PL Apo 63X /1.2). Two images were collected at each time point: monochromatic gray level and cross-polarized images. Images were acquired in a format of 1920x1440 pixels (pixel size 0.103 μm). Time lapse was set to 20 min, and Z stacks were collected using parameters that were adapted per position.

#### Image processing

Images were processed for visualization using the LasX software pack for analysis (Leica Microsystems)

### Raman spectroscopy

The cells were immobilized as follows: a pellet of natural *P*. *lenticularis* cells was mixed with a 3% low-melting agarose solution (LE agarose, HydraGene) and was loaded as a thin layer on a glass-bottom cell-culture dish. Before measurement, liquid N-HS-Ca medium was added above the solid sample. Cells were alive during the Raman measurements, and were identified using a light microscope 60X, LUMPlanFL N, NA 1.00 water-immersion objective (Olympus). Raman spectra were obtained using LabRAM HR Evolution (Horiba Scientific), a confocal micro-Raman spectrometer, using its 532 nm laser excitation and a 600 gr/mm grating with sub 2 cm^-1^ pixel resolution. Cells were photo-bleached using the 532 nm laser with 7 mW laser power for 2–10 min before mapping. The instrument utilizes an 800mm focal length spectrograph for high resolution and low stray light. The spectra underwent baseline correction and gentle Savitzky-Golay filtering using a 3^rd^ order filter with a 5 pixels window.

### Cryo-SEM

Pellets of *P*. *lenticularis* cells were high pressure frozen (HPF) (EM ICE; Leica Microsystems). The frozen samples were freeze-fractured (BAF60; Bal-Tec) before imaging and transferred to the Ultra 55 SEM (Zeiss) using a cryo-transfer chamber (VCT 100; Leica Microsystems). Samples were mounted on a cryo-stage operating at a temperature of −120°C and in a vacuum better than 5 × 10 ^−7^ mbar. Samples were etched (10 min, −110°C) and then observed under cryogenic conditions using the secondary electron InLens detector and a backscattered electron detector (BSE). The BSE detector reveals differences in atomic number; the higher the atomic number, the brighter the material appears in the image. Thus, materials with heavy atoms appear brighter than the lower atomic number C, H, O and N materials. The scanning parameters were adjusted as follows: landing voltage: 1 kV, WD = 2–3 mm, energy filter set on 360 eV. Image brightness and contrast levels were later further adjusted using Adobe Photoshop.

### Cryo-soft X-ray microscopy and spectroscopy

#### Sample preparation

2–4 ml of cultures (either control or depleted cells grown in a P-depleted medium) were transferred into a 1.5 ml Eppendorf tube and concentrated by centrifugation (310 rcf, 3–5 min). A 4 μl drop of the concentrated sample was mounted on freshly glow discharged Quantifoil S2/2 Cu grids (Quantifoil Micro Tools GmbH). A 1 μl drop of 5X concentrated gold nanoparticle (150 nm) suspension (746649; Sigma Aldrich) was added on top of the algal sample to provide fiducial markers for tomography. The grids were blotted for 3 s and plunge-frozen into liquid ethane (Leica EM-GP plunger; Leica Microsystems). Frozen grids were kept in a liquid N_2_ Dewar until transferred under cryogenic conditions into the full field soft X-ray transmission microscope where tomography and spectro-microscopy measurements of whole frozen hydrated cells were performed.

#### Data acquisition

X-ray imaging was performed at the MISTRAL beamline of the ALBA Synchrotron (Barcelona, Spain) [37].

### Cryo-soft X-ray tomography and microscopy

A tilt series at 520 eV X-ray energy was collected to allow 3D volume reconstruction of cells and their internal structures. At this energy, both C and N are highly absorbing. Each tilt series consisted of 121 to 141 images taken at 1° intervals with an exposure time of 2 s, optimizing signal-to-noise level while minimizing radiation damage. The images were acquired using a zone plate objective lens with an outermost zone width of Δrn = 40 nm, whose lateral spatial resolution was estimated to be about 30 nm half pitch. The effective pixel size in the projection images was 13.7 nm. This pixel size value enabled having a full cell in a single field of view. The projection images of the tilt series were normalized using the flat field (average of 10 images with no sample, collected at 2 s exposure time), to take into account the intensity distribution delivered to the sample by the capillary condenser lens. A Wiener deconvolution filter was applied on the projections to increase the contrast in the final reconstruction. Characterization of transfer function, resolution, and depth of field of a soft X-ray microscope was applied to tomography enhancement by Wiener deconvolution [38]. The alignment of the tomographic projections was performed in Bsoft [39] using gold nanoparticles, or dark features inside the cells, as fiducial markers. The aligned projection tilt series were reconstructed in TomoJ using the ART algorithm with 15 iterations and 0.1 relaxation coefficient [40].

### X-ray Absorbance Near Edge Spectroscopy (XANES) analysis

Energy scan series around the nitrogen K-edge of (~400 eV) were acquired by imaging the same field of view at varying X-ray energies. The acquisition procedure was as follows: in the range 389 to 397 eV with 0.5 eV-steps, 397 to 407 eV with 0.1-eV steps, and 407 to 425 eV at 0.5-eV steps. Each image was taken with 2 s exposure time. Then, all the transmission images were aligned with respect to the first image, applying the x–y shifts, which maximize the cross-correlation between the same selected r in the two images. Finally, each stack of transmission images was transformed from transmission to absorbance by taking the (–) natural logarithm of each image using ImageJ [41]. Guanine nitrogen 2D localization in the samples was carried out by subtracting an image of the cell taken at 395 eV (before the N K-edge) from the image taken at 401.4 eV (highest N K-edge absorbance), creating a map for guanine localization. The guanine maps for all cells were examined, and areas of high-absorbing pixels were identified as guanine-rich particles according to their XANES spectra. These guanine-rich areas were segmented manually.

### Data segmentation

Data were segmented using the Amira software V2021.2 (Thermo-Fisher Scientific, Eindhoven, The Netherlands). Features such as cell membranes, chloroplast and starch granules were segmented manually. Guanine crystals were segmented based on contrast and their locations were confirmed based on the energy scan data sets. Volume measurements were performed using the Label Analysis module of the AMIRA 3D software, which calculates the volume of each defined species as volume = amount of segmented voxels x voxel size. See S1 File for the link to download the data obtained from the ALBA synchrotron.

## Results

**Fig 1:**
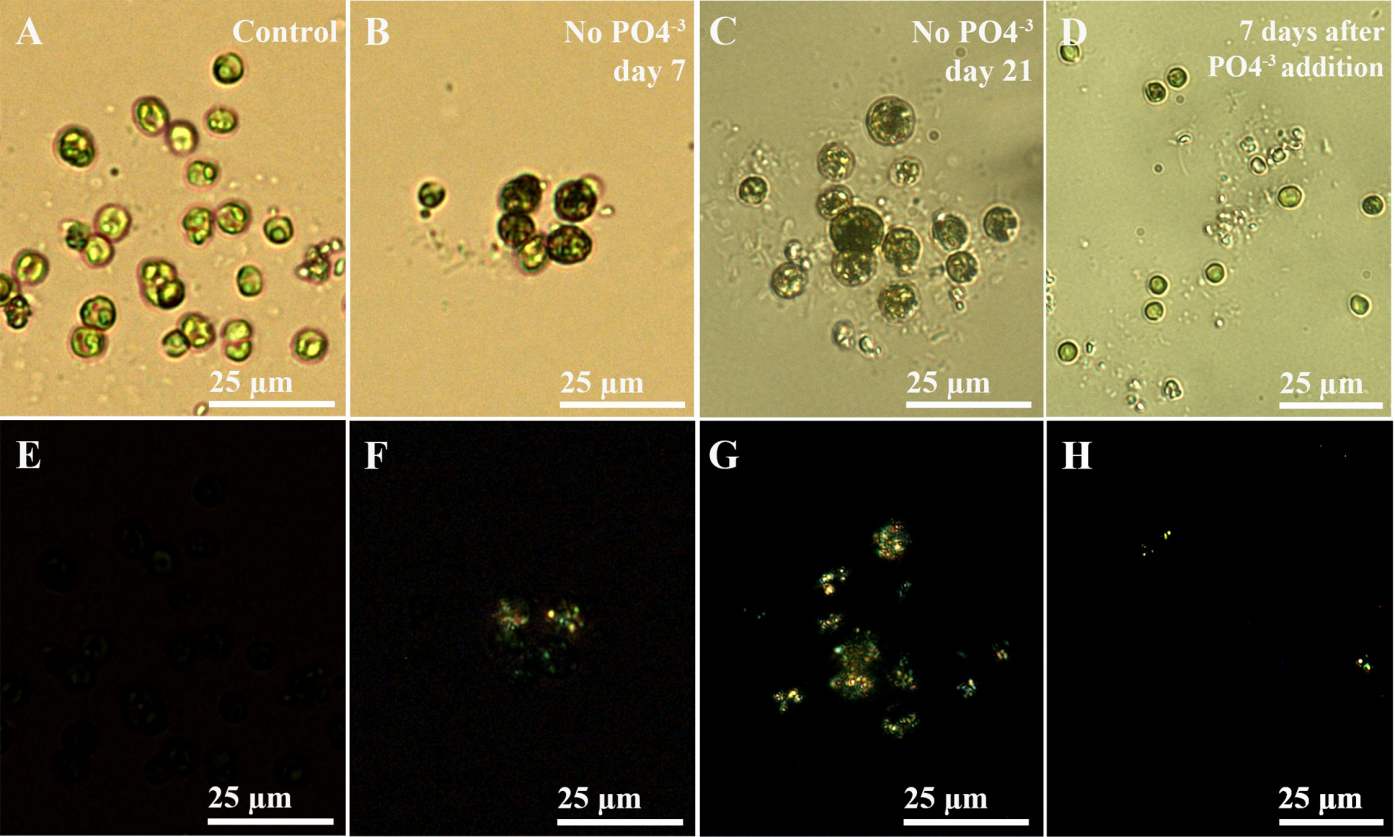
Effect of phosphate depletion on *P*. *lenticularis* strain 16.99. (A-D) Bright field images; (E-H) Cross-polarized light images; (A, E) Control culture, with no inner cellular birefringence (B, F) Algae grown for 7 days in a medium lacking phosphate, with clear intracellular birefringence. (C, G) Algae grown for 21-days in a medium lacking phosphate, showing increased size and birefringence. (D, H) The same culture as in C, 7 days after renewed phosphate supply to the medium: birefringence is significantly reduced.

Three strains of *P*. *lenticularis* cells purchased from the Culture Collection of Algae at Göttingen University (SAG) were grown in the N-HS-Ca medium adapted for the growth of *P*. *lenticularis* [35], as well as in the same medium totally depleted in phosphate. These cells did not produce under any conditions, including high Ca supersaturation, calcitic shells characteristic of the species during the bloom period. The motivation for culturing in the P-depleted medium was to determine whether this medium would trigger a nutrient-shortage response that in turn might induce calcitic shell formation. After several days of growth in the P-depleted medium all three strains of *P*. *lenticularis* (15.99, 16.99 and 61–1) produced an intracellular birefringence signal, which was observed under cross-polarized light using a petrographic light microscope (Fig 1B and 1F). The control cells in the normal medium did not have any birefringence signals (Fig 1A and 1E). After 3 weeks of growth under P-depletion conditions, all the living cells from the 3 strains had birefringence. A birefringence signal indicates the presence of periodically organized structures, and is often used for the identification of crystals. We therefore decided to investigate this phenomenon in depth using one of the three strains, namely strain 16.99, that produced birefringent inclusions after one week of P-depletion. We observed an increase in the number of cells with birefringence signals as long as phosphate depletion continued, until all the cells contained birefringent inclusions (Fig 1C and 1G). In addition, the volumes of the individual cells growing in the phosphate-depleted (P-depleted) medium increased. Notably, the cells did not produce shells during this experiment. Upon renewed supply of phosphate in the same concentrations as the original medium, the number of cells with birefringence substantially decreased, and there were no cells with a birefringence signal 7 days after P-addition (Fig 1D and 1H).

**Fig 2:**
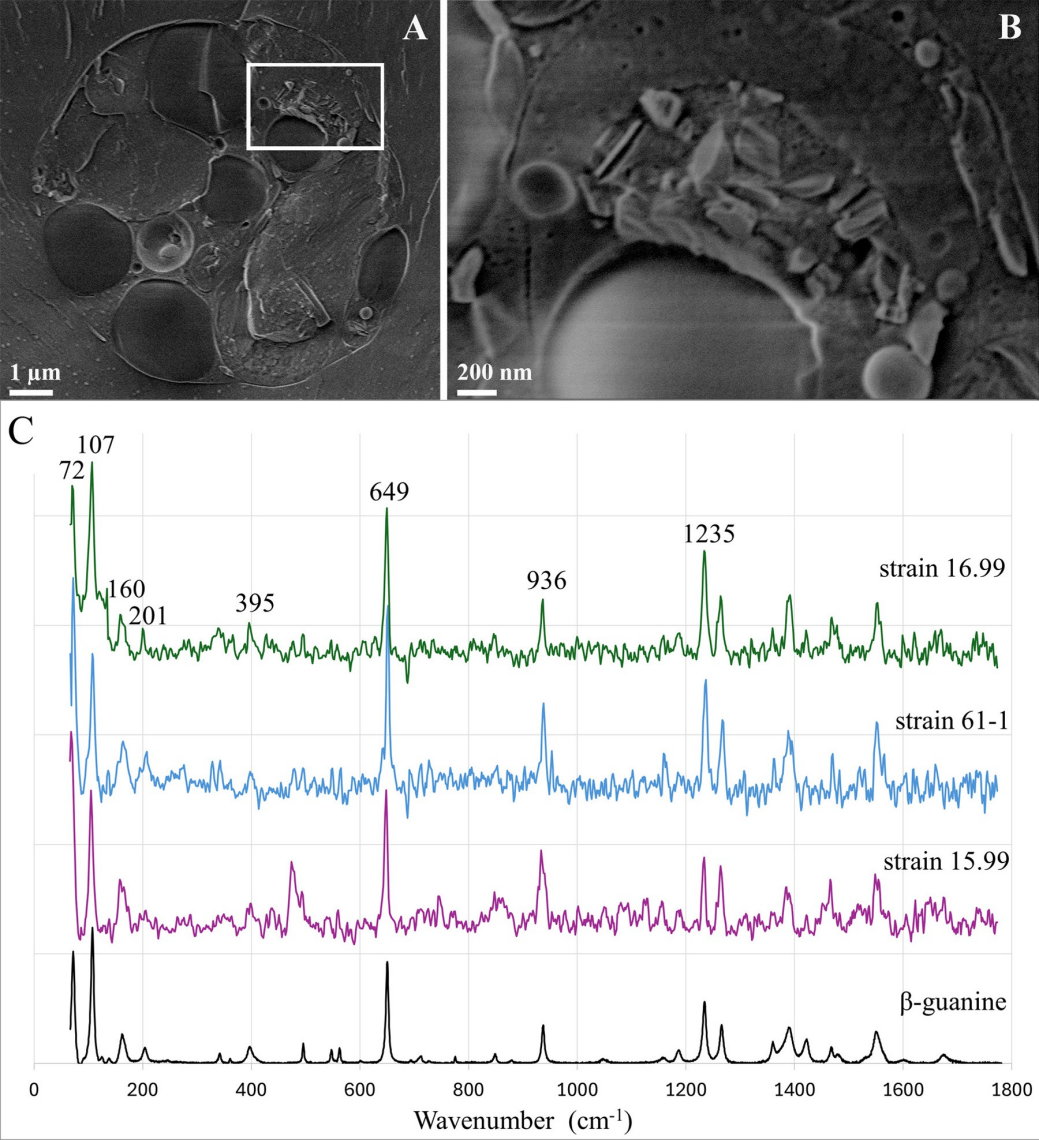
Cryo-SEM and Raman examination of birefringent crystals in *P*. *lenticularis* cells. A) A whole cell, containing many vacuoles in diverse size. (B) Enlarged observation on a single vacuole (marked with rectangle in A), containing a large amount of guanine crystals. C) Identification of birefringent crystals as guanine crystals using *in-situ* Raman spectroscopy. Reference spectrum (Bottom) and spectra collected from each of the three strains (15.99, 61–1 and 16.99 bottom to top respectively) show a correlation to guanine characteristic peaks at 650, 938 and 1235 cm^-1^. β-guanine unique polymorph characteristic peaks are also evident at 72, 107, and 201 cm^-1^.

Examination of freeze-fractured surfaces by cryo-SEM of cells with birefringence showed that crystals formed inside the cells (Fig 2A). The crystals were recognized in cryo-SEM by their characteristic faceted morphologies. The crystals were clustered inside membrane delimited compartments and were present in disorganized aggregates lacking preferred orientation (Fig 2B). The crystals did not produce any back scattering signal, raising the possibility that they were composed of organic compounds. Indeed, Raman spectroscopy of live cells from all 3 strains (15.99, 16.99 and 61–1) showed that the crystals were composed of guanine (Fig 2C). Furthermore, close examination of the spectra in the region between 50–450 cm^-1^ showed that guanine crystallized as the β-polymorph (Fig 2C).

### 3D imaging and analysis of the intracellular guanine crystals

To better understand the 3D distribution of guanine crystals within *P*. *lenticularis* cells, we examined P-depleted cells for the characteristic absorbance of guanine nitrogen under cryo-conditions using cryo-X-ray absorption near edge spectro-microscopy (XANES-microscopy).

**Fig 3:**
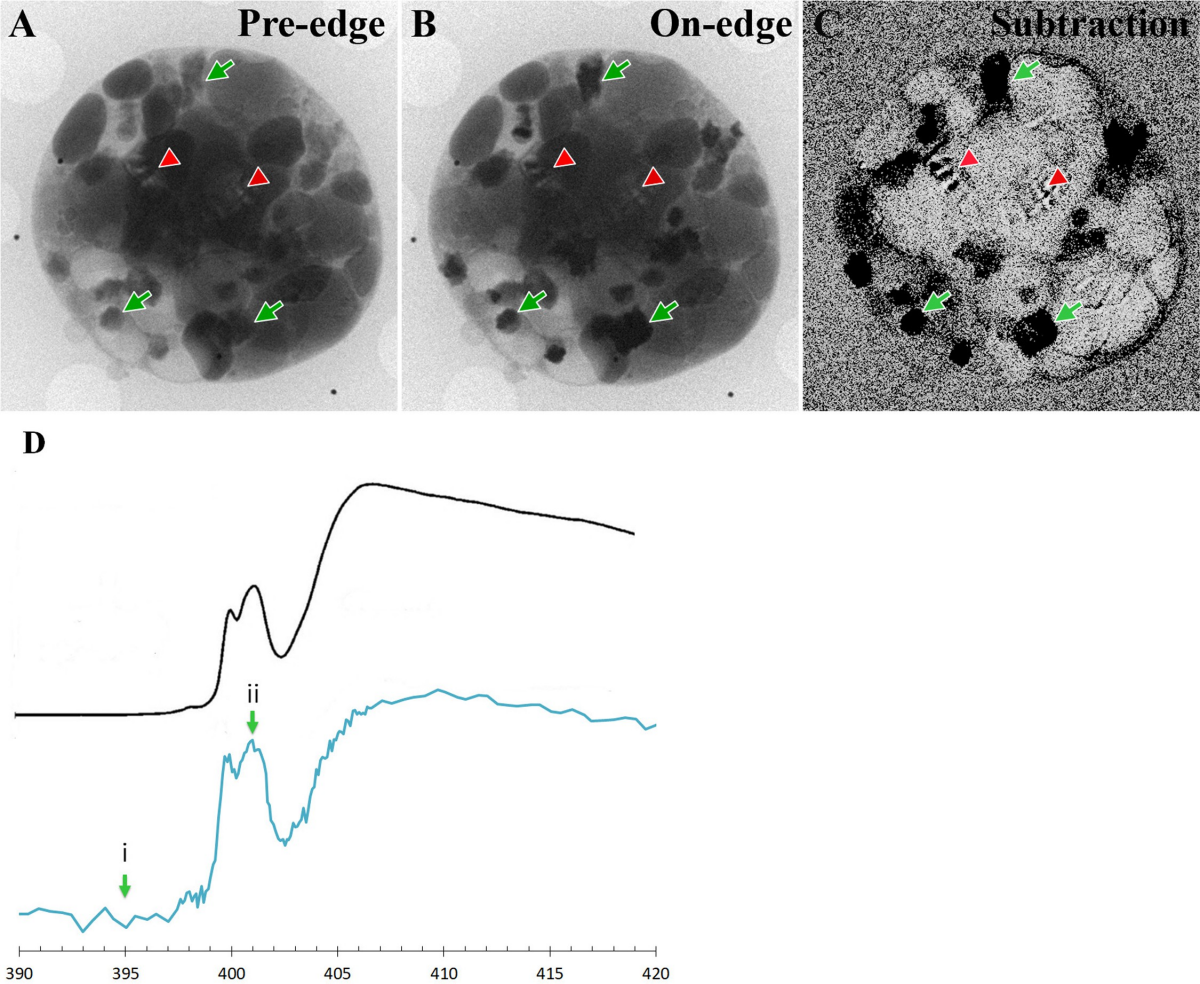
XANES measurement of a *P*. *lenticularis* cell after 2 weeks of phosphate depletion. A) Absorbance at 395eV, before the guanine-nitrogen edge. B) Absorbance at 401.4eV, on the K-edge of nitrogen, where nitrogen in guanine is highly absorbing. C) Subtraction of the two images. The black pixels (some marked with green arrows) are high nitrogen areas. The striped circle in the middle (red arrowheads) is an artifact due to stray light background [44]. D) Bottom spectrum (blue): absorption spectrum from N-rich area. Top (black) spectrum; typical spectrum of guanine crystal absorbance. Arrows i and ii mark the energy values from which Fig 3A and 3B were collected respectively. Guanine reference spectrum was reproduced with permission of the International Union of Crystallography from [43].

We used X-rays with energy within the ’water window’, where the contrast between oxygen and elements with an absorption edge in the range 280–520 eV (including carbon and nitrogen) is optimized [42]. We focused on the range corresponding to the absorption edge of nitrogen [43]. The X-rays passed through the cryo-vitrified cell and were absorbed in proportion to the nitrogen and carbon contents. A tilt series of images was reconstructed into tomograms, to produce a 3D image of the whole cell volume. Because guanine in general, and guanine crystals in particular, have a high density of nitrogen atoms, the crystal clusters were easily visible in this energy range on the oxygen-rich background of the cell. The guanine crystals were even more evident when the oxygen background is subtracted, and a differential image at the N K-edge was calculated for each cell (Fig 3A–3C). For this purpose, we subtracted the cell image recorded before the N-absorption edge (pre-edge, 395 eV) from the image recorded at the absorption edge (on-edge, 401.4 eV). Because the absorbance of all the other elements was almost the same at these two energies, the differential image represents the 2D nitrogen distribution in the corresponding field of view (Fig 3C).

**Fig 4:**
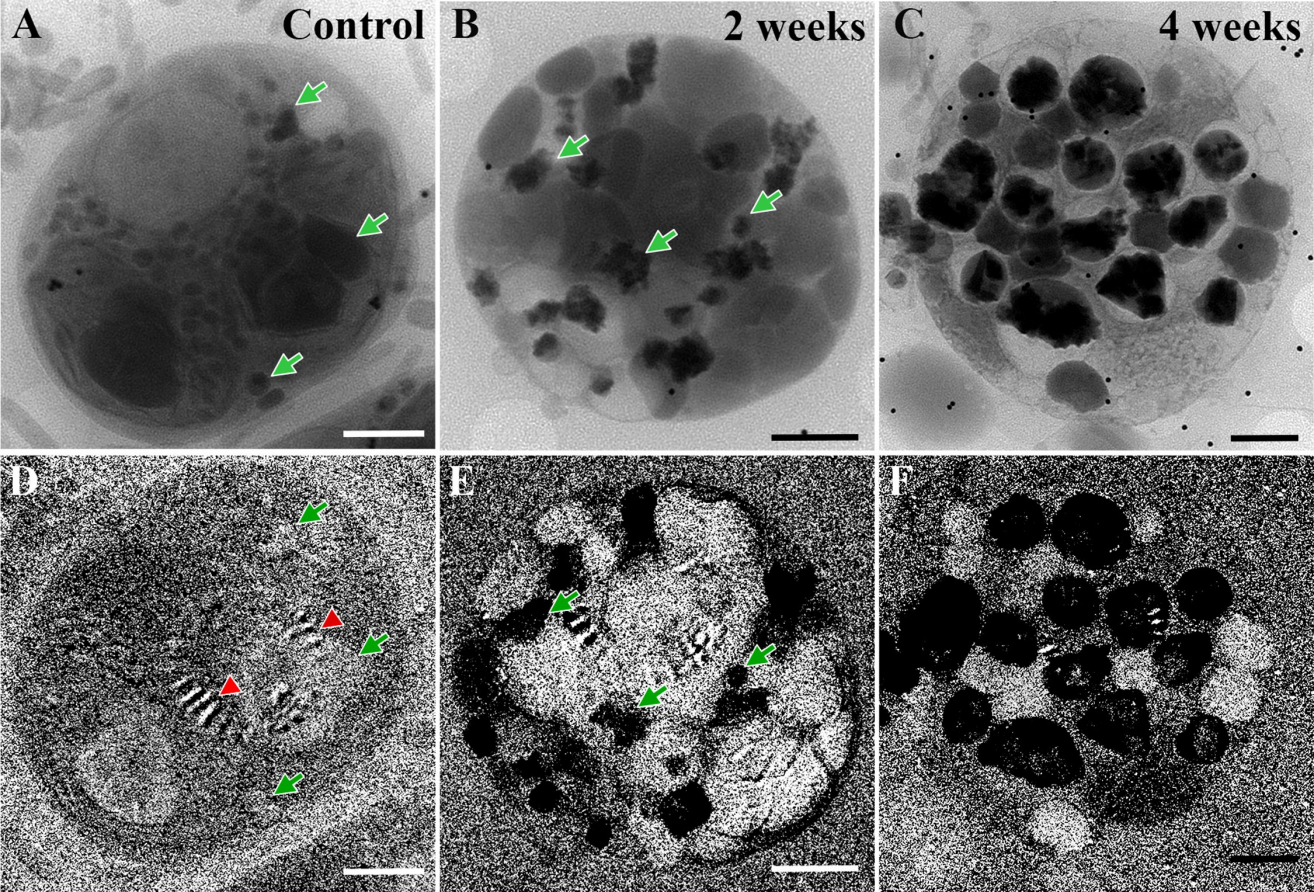
Guanine accumulation in *P*. *lenticularis* cells upon phosphate depletion. A-C) Cryo-SXT zero tilt projection images (collected at 520 eV (of A) Control cell, where no guanine crystals are present. B) 2 weeks post P-depletion, showing highly absorbing scattered guanine clusters (some are marked with arrows). C) 4 weeks post P-depletion, showing a larger amount of guanine crystals clusters. D-F) Subtracted images of the cells in A-C respectively, showing guanine crystal maps, highlighted by subtraction of the absorbance at 401.4 eV from the absorbance at 395 eV (as in Fig 3C). Green arrows mark dark features in A and B, and their location in the subtracted images D and E respectively. Notice that dark features in the control do not appear black in the crystal map on D. The red arrows mark an artifact due to stray light background, which can also be seen in E and F [44]; scale bars- 2μm.

Furthermore, for each image pixel the XANES spectrum was recorded, by scanning the absorption energy around the N K-edge (~400 eV). The spectrum of the dark regions identified by the differential image was extracted and compared to the XANES reference spectrum of guanine crystals (Fig 3D), confirming that the highly absorbing clusters (Figs 3B, 3C and 4), are guanine crystal accumulations [44].

Using cryo-soft X-ray Microscopy (cryo-SXM) images (recorded at 520 eV) we compared control cells to P-depleted cells after 2 and 4 weeks of P-depletion (Fig 4). Guanine crystals were clearly present after two weeks of P-depletion (Fig 4B and 4E) and were distributed throughout the entire cell with no specific localization (e.g. close to the cell periphery or clustered in the cell center). The accumulation of guanine crystals increased with prolonged P-depletion, until the crystals occupied a significant part of the cell after 4 weeks (Fig 4C and 4F). Observation of guanine crystals in cryo-SXM confirmed the observations from cryo-SEM, namely that the crystals vary in shape and size and do not exhibit a specific orientation.

To better understand the cellular response to P-depletion, we mapped the cell morphological changes induced by phosphate depletion after two and four weeks. We used cryo-soft X-ray Tomography (cryo-SXT) (collected at 520 eV) to reconstruct the 3D organization of 5–7 cells of each group. The observed 2D guanine crystal distribution was extended to 3D by comparison with the 2D differential imaging recorded on the same field of view. We observed the entire cell volume in 3D and characterized the cell organelles as well as the guanine crystal clusters. Four cells of each group were chosen based on the high quality of the reconstructed images, and were segmented to differentiate between the starch granules, guanine crystals and other cellular components.

**Fig 5:**
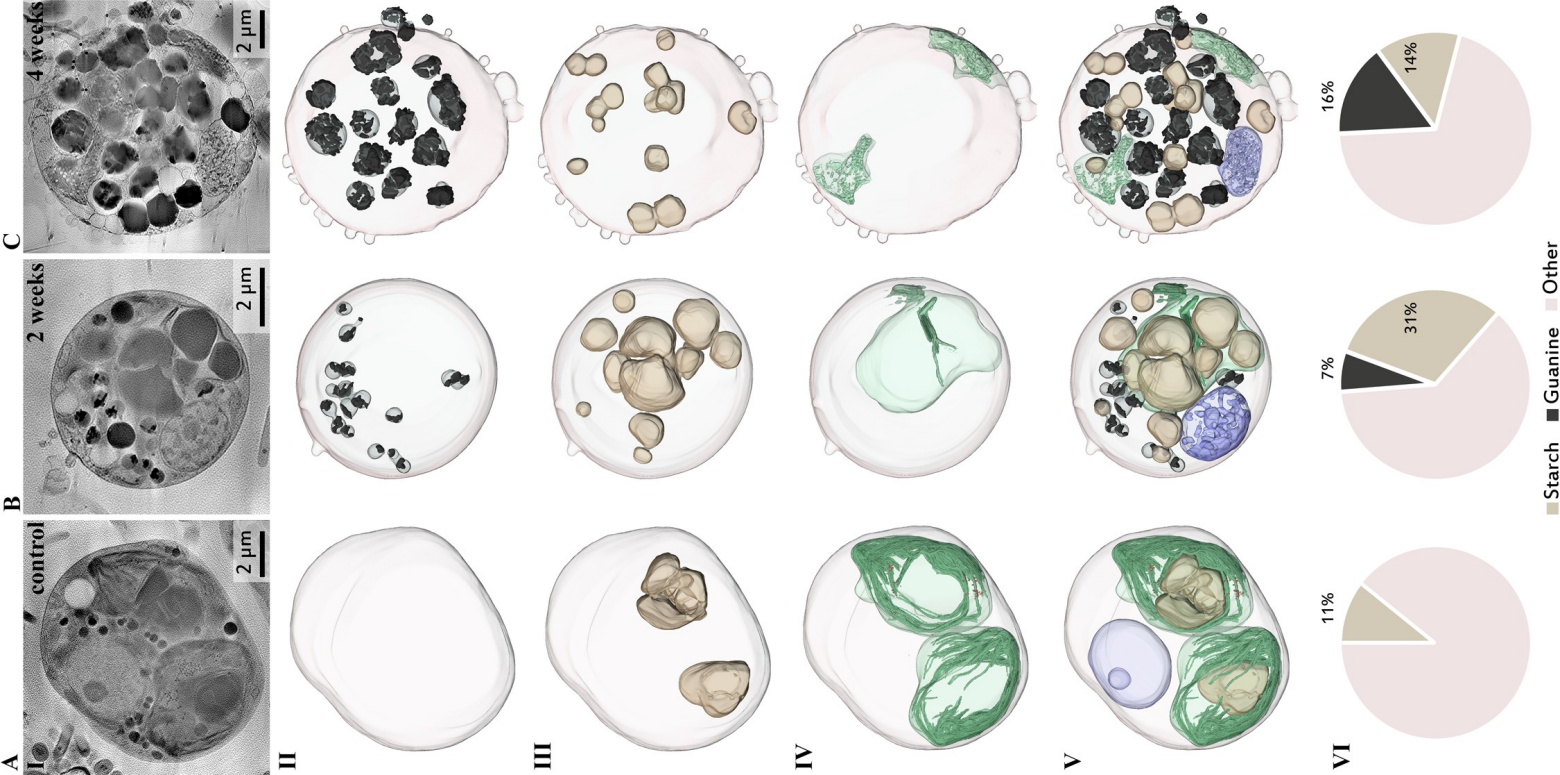
Surface representation and volume analysis of *P*. *lenticularis* segmented cells based on cryo-soft X-ray tomography. Columns: A) Control, B) after 2 weeks of phosphate depletion and C) after 4 weeks of phosphate depletion. Guanine crystals (black), starch granules (beige) and chloroplast (green) are segmented and compared between control, 2-week, and 4-week P-depleted cells. Rows: I) Average of several slices taken from the middle of the reconstructed cell volume. II) Surface representation of the guanine crystal aggregate fraction in the cell (black). III) Surface representation of the starch fraction (beige). IV) Surface representation of the chloroplast structures (green) V) Merge of guanine, starch, and chloroplast fractions, combined with the nucleus (purple). VI) Volume analysis of 4 cells of each kind, showing the proportional volume occupied by guanine clusters and starch granules in each type of cells. Note that the scale bars in Row I are not identical and that the cells in Column B are smaller than in columns A and C.

Massive changes to the entire cell structure were caused by P-depletion. The guanine crystals, which were absent in the control cells (Fig 5AI and 5AII), occupied 7±2% of the cellular volume after 2 weeks of P-depletion (Fig 5BI, 5BII and 5BVI), and 16±6% of the cellular volume after 4 weeks of P-depletion (Fig 5CI, 5CIII and 5CIVI). Control cells contained large chloroplasts that filled almost the entire cell and contained one or two large starch granules (Fig 5AIII and 5AIV). In the P-depleted cells after 2 weeks, the chloroplasts appeared to be degraded, and the cells were mostly filled by starch granules (Fig 5BIII and 5BIV). These starch granules varied in size and occupied 31±7% of the cell volume, which is a significantly larger part of the cell volume relative to the control (11±1%) (Fig 5AIII, 5AV, 5BIII and 5BV). After 4 weeks of P-depletion, the chloroplasts were deformed and were no longer filled by starch granules (Fig 5C and 5CIV). Instead, starch granules were distributed in the cell volume, with no apparent surrounding membrane (Fig 5CIII). The volume of starch granules decreased by half in comparison to the 2-week P-depleted cells (14±7%).

**Fig 6:**
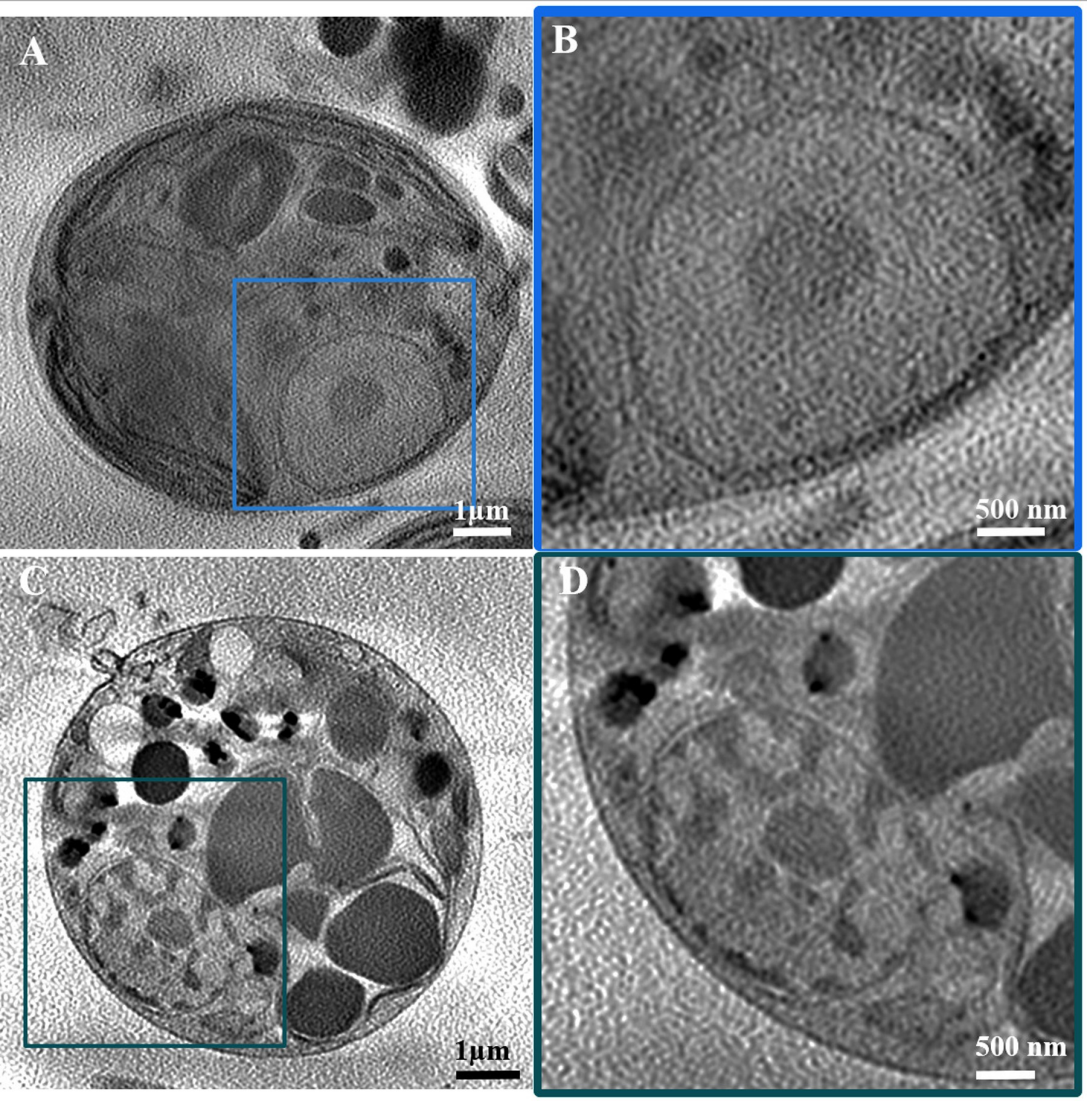
DNA inhomogeneity in the nucleus of phosphate-depleted cells. A) Control cell from 2 weeks old culture. B) High magnification of the cell nucleus, showing the clear nucleolus inside. C) Phosphate-depleted cell at the same time point, showing major changes to the nucleus organization, indicative of chromatin condensation. D) Higher magnification of the nucleus from the blue frame in C).

An additional change in the cellular organelles was observed in the nucleus organization. In contrast to control cells, where the nucleus volume had a homogeneous appearance, and contained only a distinct nucleolus area in its center (Fig 6A and 6B), 2-weeks P-depleted cells had an inhomogeneous nucleus, with irregular densities (Fig 6C and 6D). The nucleolus was still evident, but its shape was less coherent (Fig 6D). This observation is consistent with chromatin condensation within the nucleus.

**Fig 7:**
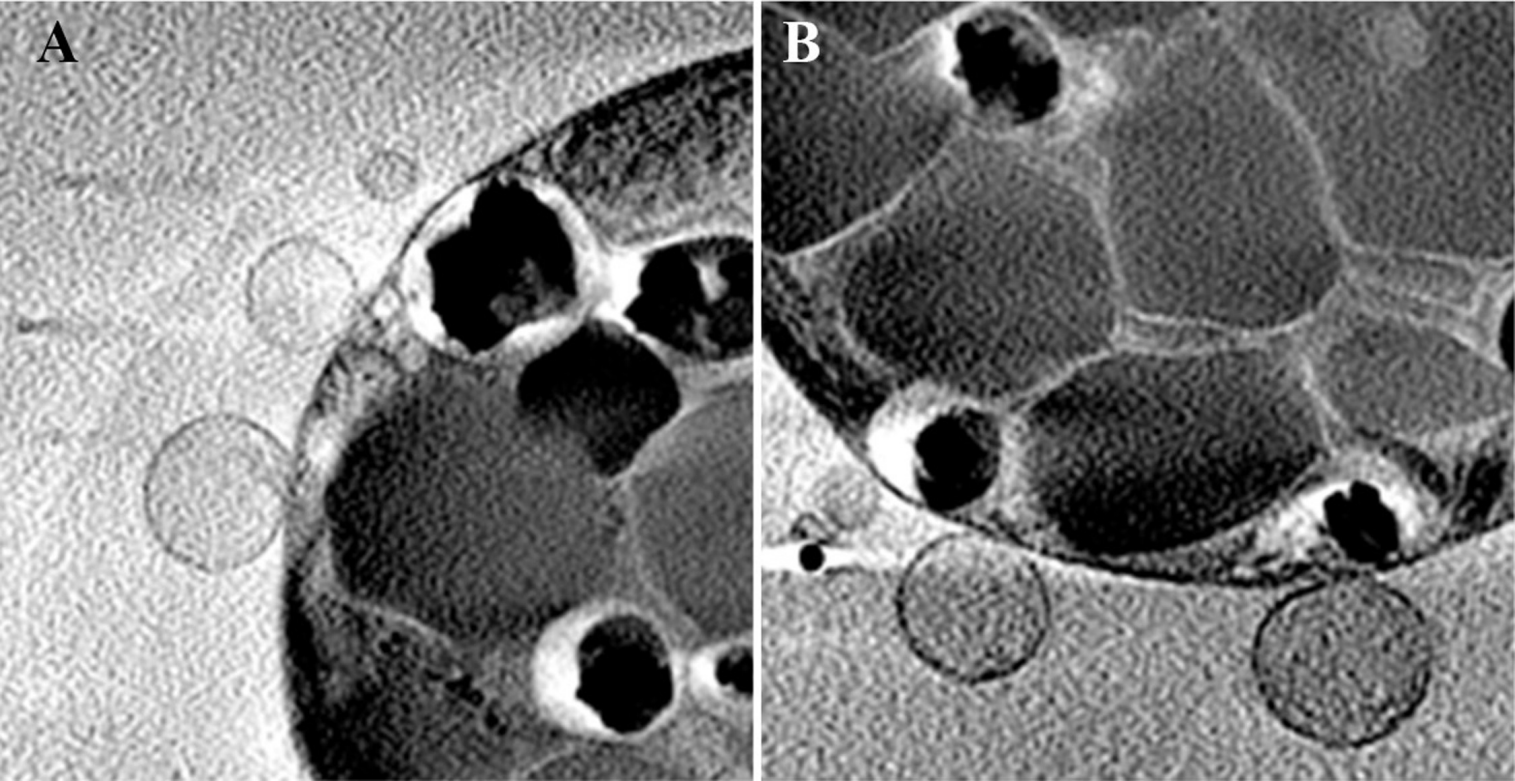
Membrane blebbing leading to volume change in two areas of a P-depleted cell. A) Top left quarter of the cell. B) Bottom of the same cell.

In addition to the above cell morphology changes, cells grown in P-depleted medium exhibited membranal blebbing (Fig 7). This was in contrast to the control cells, where no blebbing was observed. We also observed massive changes in cell volume. While the average volume of control cells was about 190 μm^3^, the volume of cells after 2 weeks in P-depleted medium decreased by almost half. This observation is consistent with the cellular blebbing. At 4 weeks of phosphate depletion, some of the cells swelled, leading to an average volume of 140 μm^3^, about 75±24% of the control original volume.

**Fig 8:**
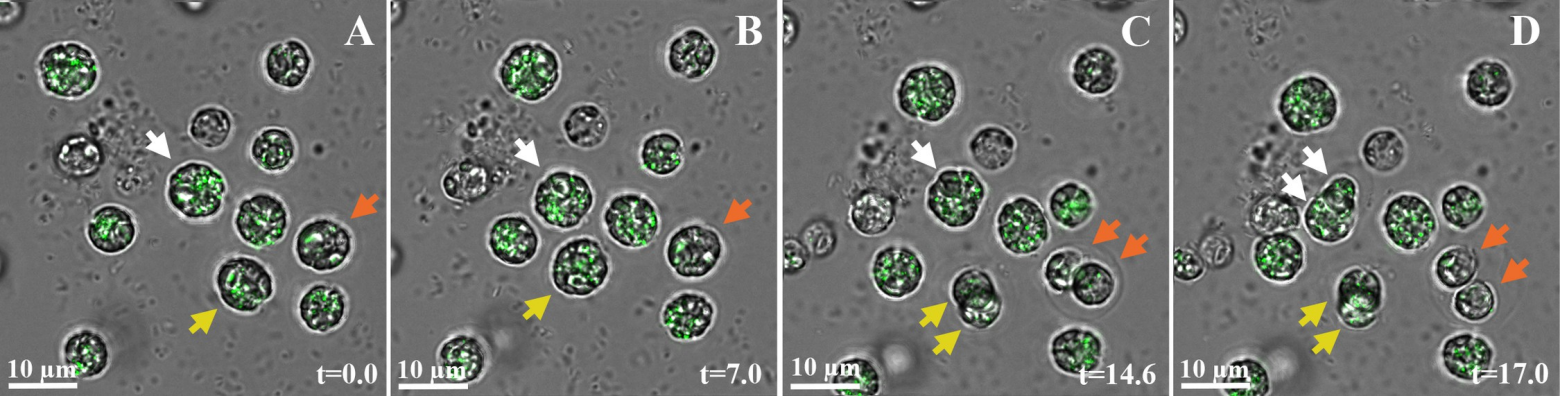
*P*. *lenticularis* cells recovery after phosphate addition. In all images, the monochromatic image (grey levels) is overlayed on the polarization signal (green). A) *P*. *lenticularis* cells after 19 days of P-depletion, immediately after change to a normal medium. Three of the cells, containing significant amount of crystals, are marked with arrows (white, yellow, and orange) to facilitate tracking them in images B-D. B) The same group of cells after 7h of incubation in P-containing medium. Note that the cells are not attached to the bottom and thus move slightly. C) 14.6h post P-addition, two of the cells reproduced. Daughter cells are marked by two arrows of the respective color (orange and yellow arrows). D) 17h post P-addition, an additional cell reproduced (white arrows).

All the morphological changes described above occurred while the cells were grown in protracted P-depletion conditions. We then addressed the question of what happens to these P-depleted cells when phosphate is added back into the medium. The cells were continuously monitored for 24h post phosphate addition using a time-lapse light microscope set up (Fig 8). Cells containing multiple guanine crystals (Fig 8A and 8B) began reproducing 10h after phosphate addition (S1 Video), and continued to reproduce without apparent impairment. In contrast, the few cells in the P-depleted medium that did not contain guanine crystals, did not reproduce. Daughter cell volumes are smaller following cell division, such that the amount of birefringent signal is reduced, but it is difficult to evaluate if the total amount of crystals in the daughter cells is reduced relative to the mother cell.

## Discussion

Here we show that cultured strains of *P*. *lenticularis* that do not produce calcitic shells in standard media, do produce intracellular guanine crystals when the medium is depleted in phosphate (P). In the P-depleted medium the cells also show a reduction in chloroplast volume, an increase in starch granule volume, nuclear DNA condensation and membrane blebbing. All these changes are consistent with the depletion of P inducing a stress response in the *P*. *lenticularis* cells. Upon reintroduction of phosphate, the cells resume their natural pattern of reproduction notwithstanding the conspicuous presence of the guanine crystals. Clearly, the guanine crystals do not impact cell recovery, but possibly support cell recovery. We therefore propose that the formation of intracellular guanine crystals is also part of the stress response.

Stress response in plants can be induced by many abiotic conditions such as high salinity, change in light exposure, temperature flux, UV radiation and nutrient deficiency. To overcome the stressful conditions, organisms, including algae, activate mechanisms to support their survival in the changing environment [45, 46]. Stress response-induced changes in cell structure and morphology are more evident when observing a single-cell organism. Thus, many genetic and morphological studies were conducted to record stress response mechanisms in unicellular species.

Significant morphological changes are observed in green algae under different stress conditions. Responses to high salinity changes often include slowing down of cell division, reduction in cell size, flagellar and chloroplast degradation, and increased secretion of exopolysaccharides, meant to facilitate the formation of algal colonies of non-motile cells embedded in a matrix (palmelloid) [47]. Shortage of light can trigger changes in cell volume and changes in the number of thylakoids in the chloroplast [48, 49]. Shortage of light also induces modifications in photosynthetic activity, leading to changes in the cell cycle rate and accumulation of damaging oxides [50]. Exposure to ultraviolet (UV) radiation often damages photosynthesis and leads to slower reproduction, while DNA repair mechanisms are upregulated [51, 52]. Nutrient deficiency (N/P/S) often triggers cellular changes and the accumulation of starch granules, followed by its reduction when the deficiency is prolonged [53–55]. Growth rate decreases during nutrient deficiency, and levels of photosynthesis and respiration are also reduced [56]. Notably, prolonged nutrient shortage can lead to reduced viability and cell death.

Our observations in *P*. *lenticularis*, and specifically the accumulation of starch granules, the reduction of chloroplast volume and the changes in cell volume, are commonly observed in many algal cellular-stress responses (as summarized in the previous section, [57, 58]). On the other hand, changes in nucleus morphology and DNA condensation have not been related to algal stress. Chromatin condensation in algae, as well as in plants, is often related to programmed cell death (PCD) by apoptosis, which may be the result of prolonged stress. We emphasize that the DNA condensation observed here in *P*. *lenticularis*, as well as other morphological changes, including guanine crystal formation, are not PCD-related, because cell recovery occurs in the general population (Fig 1), and specifically in the cells containing crystals (Fig 8).

Together, the observations presented in this study show that *P*. *lenticularis*, like most photosynthetic organisms, is sensitive to phosphate levels in the environment, and especially to phosphate shortage. The lack of phosphate triggers a massive cellular response, which is evident when observing the changes in different organelles. As guanine crystal formation in *P*. *lenticularis* cells occurs in parallel to the documented overall stress response, we infer that the formation of guanine crystals is also a part of this general cellular stress response.

*P*. *lenticularis* algae live in freshwater basins, which generally undergo extreme seasonal changes. Under optimal conditions the *P*. *lenticularis* cells bloom, and when the conditions become less optimal, they “disappear”, either due to the small number of cells in the basin or because the cells do not produce shells and are therefore unrecognizable. Thus, the life cycle of *P*. *lenticularis* must somehow be adapted to stress. We were unable to determine whether or not the wild type cells living in an ephemeral small basin of water in Israel [32] produce intracellular guanine crystals under stress, because the presence of the shell masks the possible detection of the guanine crystals. The *Phacotacea* are distinguished by the presence of a lorica, but *P*. *lenticularis* can only be distinguished from the other unicellular green algae present in the environment by the presence of a two-shelled mineralized lorica [59].

The accumulation of nitrogen (N) within algal cells was observed in several studies [24, 25, 60, 61]. In all, N-rich vacuoles were observed following an intentional exposure to stress conditions. Shebanova et al [60] induced either P or N shortage, combined with limiting CO_2_ supply. Moudříková et al. [24] and Mojzeš et al. [61] induced the formation of guanine crystals by N-starvation followed by addition of a nitrogen source in limited CO_2_ environment. Pilatova et al. [25] also observed guanine crystallization when culturing cells in medium providing an abundance of N. These conditions can trigger a stress response due to high (and possibly toxic) nitrate or ammonia concentrations.

Here, we report the formation of guanine crystals following P-depletion, which is of particular interest relative to the research discussed above, as phosphorus is not part of the guanine molecule, and thus guanine crystals cannot provide a means for phosphorus storage. It is not surprising that phosphate depletion triggers a stress response in the cell, as phosphate is fundamental to many cellular building blocks, such as phospholipids, nucleotides and low-molecular weight water-soluble phosphate-esters and anhydrides [53, 62]. Phosphate is also stored in the cell, usually in the form of poly-phosphate-containing vacuoles [60, 62, 63]. Interestingly, unlike in the N-depletion experiments, where guanine crystals form after restoring N-supply, we see here the accumulation of guanine crystals immediately after inducing P-depletion. This shows that the stress response mechanisms are very rapid and activated immediately after the shortage is sensed by the algal cell. We believe that the rapid accumulation of guanine crystals in our observations is attributable to the fact that N was available to the cells during the stress induction, as the depleted nutrient is phosphate.

Interestingly, Pilatova et al. [25], observed guanine crystal accumulation in unicellular eukaryotic species taken directly from culture or from the natural environment. This raises the possibility that what was originally a stress response may have evolved into an integrated function of the cells. Pilatova et al. [25] also showed that the formation of guanine crystals in unicellular organisms is a widespread phenomenon in eukaryotes [25]. A more recent study by Pavan et al. [64] showed that the phenomenon extends even to some prokaryotes, supporting the possibility that guanine crystal formation is functional.

Several possible explanations were proposed for the function of guanine crystals, including bioluminescence [19, 20] and detoxification following the exposure to high ammonia or nitrate levels [22]. In the more recent studies, involving nitrogen depletion, the function of guanine crystals was inferred to be nitrogen storage, following the sensing of carbon-nitrogen imbalance in the immediate environment [25, 60, 61]. As most microalgae are at some point exposed to low-nutrient environment, a stress response that triggers storage of essential nutrients is advantageous for cell survival. Thus, we propose that guanine crystallization is part of a generic response to stress, where the cell accumulates essential nutrients, including nitrogen, carbon (as starch) and possibly other nutrients such as phosphorus [60]. As phosphorus, nitrogen and carbon are important to all unicellular organisms, it may explain why this phenomenon is so widespread across unicellular organisms.

## Conclusions

We show that guanine crystal formation can be induced following the depletion not of nitrogen, but rather of the equally essential nutrient source, phosphorus. We show that in addition to guanine crystal formation, P-depletion induces a massive stress response of the cell, including accumulation of starch granules, the reduction of chloroplast volume and changes in cell volume. Moreover, upon phosphate addition, the algal cells recover, and begin to reproduce with their guanine crystal-containing vacuoles. Thus, we conclude that the guanine crystals in *P*. *lenticularis*, and possibly in other unicellular eukaryotes, are part of a generic cellular response to stress.

## Supporting information

S1 Video 19 days P-depleted *P*. *lenticularis* cells recover after phosphate addition. The cells were continuously monitored using a light microscope set up. Single time points are seen in Fig 8. Monochromatic bright field signal (grey levels) is overlayed with the polarization signal (green). The cells are not attached to the bottom and thus move slightly during the first phase of recovery. The first cell reproduces after 10h, and two more cells reproduce sequentially, still containing at least some of the crystals.
(MP4)

S1 File (DOCX)
