# Supplementary material for: Guanine crystal formation by the unicellular organism Phacotus lenticularis is part of a cellular stress response
Source: PLoS One. 2025 Feb 12;20(2):e0316193. doi: 10.1371/journal.pone.0316193 (PMC11819609; doi:10.1371/journal.pone.0316193)
Supplement: S1 File — (DOCX) [file pone.0316193.s002.docx]

**Supplementary information**

24h time-laps experiment observing the recovery of *P. lenticularis* cells post P-addition to the growing medium. The cells were continuously monitored using a light microscope set up. Single time points are seen in Fig. 8.

**Video S1:** 19 days P-depleted *P. lenticularis* cells recover after phosphate addition. Monochromatic bright field signal (grey levels) is overlayed with the polarization signal (green). The cells are not attached to the bottom and thus move slightly during the first phase of recovery. The first cell reproduces after 10h, and two more cells reproduce sequentially, still containing at least some of the crystals.

**Dataset:** the data acquired at the ALBA synchrotron may be downloaded at the following link:

The DOI for the dataset is 10.34933/f4bfb030-82bf-4088-8908-8eca709d41db

[**https://doi.org/10.34933/f4bfb030-82bf-4088-8908-8eca709d41db**](https://doi.org/10.34933/f4bfb030-82bf-4088-8908-8eca709d41db)
